# Surgical Anatomy

**Published:** 1852-06

**Authors:** 


*Surgical Anatomy.* By Joseph Maclise, Surgeon. With sixty-eight colored Plates. Philadelphia: Blanchard & Lea. 1851. Complete in five parts; price nine dollars. For sale by Derby, 164 Main-st., Buffalo.

We have received the concluding numbers of this excellent work, and we can assure our readers there is no falling off in the value of the matter, or in the style of the execution, and so much cannot always be said of a serial.

We are confirmed, therefore, in the opinion which we expressed when the first No. was laid before us, that "it is one of the best and cheapest anatomico-surgical works ever published by the American press." F. H. H.

